# Representation of published core outcome sets for research in regulatory guidance: protocol

**DOI:** 10.12688/hrbopenres.13139.2

**Published:** 2021-06-09

**Authors:** Susanna Dodd, Rebecca Fish, Sarah Gorst, Deborah Hall, Pamela Jacobsen, Jamie Kirkham, Barry Main, Karen Matvienko-Sikar, Ian J. Saldanha, Dominic Trépel, Paula R. Williamson

**Affiliations:** 1 MRC/NIHR Trials Methodology Research Partnership, Department of Health Data Science, University of Liverpool (a member of Liverpool Health Partners), Liverpool, UK; 2 Division of Cancer Sciences, University of Manchester, Manchester, UK; 3 Colorectal and Peritoneal Oncology Centre, The Christie NHS Foundation Trust, Manchester, UK; 4 Hearing Sciences, Division of Clinical Neuroscience, School of Medicine, University of Nottingham, Nottingham, UK; 5 School of Social Sciences, Heriot-Watt University Malaysia, Putrajaya, Malaysia; 6 Department of Psychology, University of Bath, Bath, UK; 7 Centre for Biostatistics, Manchester Academic Health Science Centre, University of Manchester, Manchester, UK; 8 University of Bristol Medical School, Centre for Surgical Research, Bristol, UK; 9 School of Public Health, University College Cork, Cork, Ireland; 10 Center for Evidence Synthesis in Health; Department of Health Services, Policy, and Practice, Brown University School of Public Health, Providence, Rhode Island, USA; 11 Global Brain Health Institute, Trinity College Dublin, Dublin, Ireland; 12 School of Medicine, Trinity College Dublin, Dublin, Ireland; 13 University California, San Francisco, California, USA

**Keywords:** core outcome sets, regulatory guidance

## Abstract

**Background: **The COMET Initiative promotes the development and use of ‘core outcome sets’ (COS), agreed standardised sets of outcomes that should be measured and reported in all studies in a particular clinical condition. COS are determined by consensus amongst key stakeholders, including health professionals, policymakers and patients, ensuring that the priorities and expertise of these representatives inform the choice of the most important outcomes to measure for a given condition. There is increased recognition of the need to integrate COS across the healthcare system and with existing regulatory apparatus, to ensure that outcomes being recorded are those of key relevance to important stakeholders. The aim of this study is to assess the degree of concordance between outcomes recommended in COS for research and in guidance provided by two key regulators: US Food and Drug Administration (FDA) and the European Medicines Agency (EMA).

**Methods: **COS for research published during 2015-2019 with patient involvement and covering drug or device interventions will be compared against relevant regulatory guidelines, matched by condition. Guidance documents matching in scope (relating to intervention and population) to a COS for research will be scrutinised to identify all suggested outcomes for comparison against the core outcomes in the corresponding COS.

**Discussion: **This study will identify variation between outcomes suggested in FDA and EMA regulatory guidance relative to outcomes included in published COS for research, thus demonstrating the degree of representation of COS in regulatory guidance and vice versa. We will share the study findings (in particular, highlighting any lack of concordance between COS and regulatory guidance overall or for particular disease areas) and will invite feedback from FDA and EMA; we will seek to highlight where findings support the recommendations towards using well-developed COS or will make recommendations to COS developers on outcomes of importance to these key regulators.

## Introduction

Measuring patient health outcomes helps to inform healthcare decisions that are made by patients, healthcare professionals and funders. The Core Outcome Measures in Effectiveness Trials (COMET) Initiative ^1, 2^ brings together people and groups interested in the development and application of agreed standardised sets of outcomes, known as ‘‘core outcome sets’’ (COS). One of the successes of COMET has been the development of a publicly available searchable database of completed and ongoing COS development projects ^3^. COS may be developed for research or clinical practice, and are determined by consensus amongst health professionals, researchers, policymakers and patients or their representatives, thus ensuring the priorities and expertise of these key stakeholders determine the most important outcomes to measure for a given condition. COS are increasingly being recommended for use by trial funders and healthcare organisations ^4^. The Core Outcome Set-STAndards for Development (COS-STAD) ^5^ minimum standards was published in 2017, providing benchmarks against which to assess the quality of COS. COS-STAD covers 11 key features of COS development relating to three aspects of the COS development process: scope (health condition, population and intervention covered by the COS), stakeholder involvement (including patients, healthcare professionals and researchers) and consensus process (relating to the initial outcomes lists, scoring and consensus decisions, and unambiguous wording of outcomes).

Healthcare regulators play an important role in quality improvement, and frameworks adopted by certain organisations rely on evidence on outcomes to inform decision-making ^6– 8^. Specifically, as an example, to support improvement in healthcare services in the UK, bodies such as the Healthcare Quality Improvement Partnership (HQIP) or UK National Institute for Health and Care Excellence (NICE), are recognising the relevance of considering COS for consistent measurements to inform their guidance ^9, 10^. In 2018, NICE guidance on methods to determine relevant guideline outcomes was updated to indicate that COS should be used, if suitable based on quality and validity ^9^. The HQIP tool describing key features of national clinical audits and registries states that the rationale for quality improvement objectives should take into account relevant evidence from the COMET database ^10^. Similarly, the Swedish Agency for Health Technology Assessment and Assessment of Social Services (SBU) ^11^ endorse the use of well-developed COS to inform choice of outcomes in trials and systematic reviews.

A number of research funding agencies (particularly those commissioning the use of pragmatic randomised control trial to inform policy and regulation) are increasingly recommending that applicants should consider using a COS if one exists ^4^. For example, the international SPIRIT reporting guidelines endorse consulting the COMET database to identify relevant COS ^12, 13^, and in the UK, as an example, National Institute for Health Research Health Technology Assessment (NIHR HTA) programme refers applicants to the COMET database, suggesting that they include established core outcomes “unless there is good reason to do otherwise” ^14^. The authors believe that uniformity in recommendations from NIHR and other public funders regarding use of COS would promote greater consistency in outcome collection globally. This benefit would have additional impact if the consistency in such recommendations extended to those from commercial sponsors. The NIHR is a unique health funding agency as Technology Assessment Review teams are funded to provide NICE with independent research to inform their guidance committees ^15^. Whilst the relationship between funding research and regulating health varies internationally, regulators such as the US Food and Drug Administration (FDA) and European Medicines Agency (EMA) are influential in commissioning of research to help inform their decisions and it is important to assess the degree of concordance between patient outcomes suggested in FDA and EMA guidance and core outcomes included within COS for research, matched by condition.

The US FDA publishes official Guidance Documents and other regulatory guidance ^16^, covering topics such as biologics, drugs, medical devices and food, as well as general guidance on study design and outcomes, such as their guidance on the conduct of randomised trials during the COVID-19 pandemic ^17^ or on Patient-Reported Outcome Measures. ^18^. These guidance documents describe the FDA’s current opinion on regulatory issues but are not legally binding (unlike FDA regulations, which are federal laws ^19^. Similarly, the EMA publishes scientific guidelines prepared in consultation with regulatory authorities in the European Union Member States to inform marketing authorisation applications for human medicines ^20^, with full justification required for any deviations from these guidelines. This study will compare the outcomes suggested in these guidance documents against core outcomes included within COS, matched by condition, in order to progress this field by furthering our understanding of the similarities and the differences between COS and guidelines ^21^.

## Methods

### Search strategy

**Figure 1.**
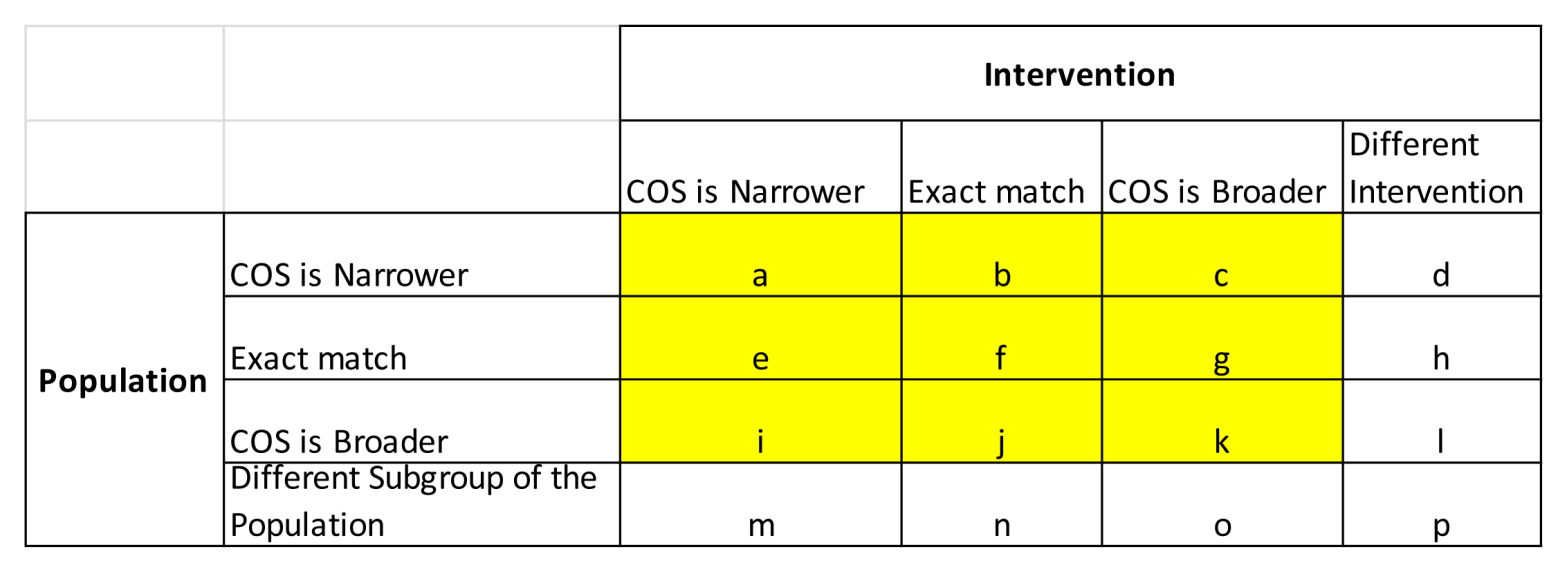
Scope matching algorithm determined according to the descriptions of the population and intervention within the FDA/EMA guideline versus the corresponding COS.

The COMET database contains 108 COS for research published between 2015 and 2019 which involved patients in the consensus process. Selection of only those COS published in the last five years which involved patients will increase the number of COS-STAD (Core Outcome Set-STAndards for Development ^5^) standards met. (Note that this study includes COS identified as part of the annual COMET systematic reviews up to and including the systematic review conducted in 2020, which only included studies published up to the end of 2019.) The scope of the COS meeting these criteria will be assessed to ensure that they cover drugs or devices, and if not, they will be excluded from the cohort. For each COS for research, we will search the FDA ^16^ and EMA ^20^ websites to identify guidance covering the relevant disease/condition, using the key clinical terms as search terms. If necessary, we will refine these searches using the Google site-specific search facility e.g. searching for “diabetes site: fda.gov” or “diabetes site: ema.europa.eu” when searching for guidelines relating to diabetes on the FDA or EMA websites respectively. We will engage with COS developers if clinical input is required for guidance on appropriate search terms (e.g. to determine synonymous clinical terms to those used to describe the disease/condition under investigation in the COS) or if there is any ambiguity regarding whether identified guidance documents match the scope of the COS (in terms of the disease/condition or interventions). We will initially identify regulatory guidance/COS pairs where the scope is an exact match but will also consider situations where one of the pair may be more general than the other, based on an assessment of the descriptions of the population (i.e. clinical condition/disease) and intervention in the COS publications and regulatory guideline documents, using a previously-developed framework ^22^ (see Figure 1). Pairs which focused on different interventions or different populations were not considered to be a match (i.e. only matches corresponding to types *a-c*. *e-g*, *i-k* in Figure 1 were eligible for inclusion). Each reviewer independently applied this matching algorithm to each pair of FDA/EMA guideline and corresponding potentially relevant COS. Discrepancies were resolved through discussion.

### Eligibility

- COS eligibility: COS for research (including those intended for both research and practice) were included if published between 2015 and 2019, involved patients in the consensus process and related to drug or device interventions.
- Regulatory guidance eligibility: EMA/FDA guidance were considered eligible for comparison against relevant COS for research if their scope (in terms of clinical condition/disease and intervention) matched at least generally with that of the corresponding COS for research (i.e. matches of type *a-c*. *e-g*, *i-k* in Figure 1).

### Data extraction

Data on the year of publication, disease name, specific condition and outcomes included in the eligible published COS for research will be exported from the COMET database and COS database into an Excel spreadsheet. We will record whether the COS developers consulted FDA and/or EMA guidelines as part of the COS development process, as detailed in the COS publication. We will also record from the COMET database whether participants from low/middle-income countries (LMIC) were involved in COS development. Once FDA and EMA guidance documents are identified which match in scope to a COS for research, the guidance documents will be scrutinised in order to identify all suggested outcomes (i.e. those outcomes which the guidelines state should/could/may/might be considered) relating to the specific COS population and intervention. Any additional caveats included in the guidelines about each of the recommended outcomes (e.g. relating to the age category or severity) will be recorded. If specific measurement tools (e.g. quality of life questionnaires) are recommended, the reviewer will search for and extract the individual items within these measurement tools, in order to assess whether these individual items correspond to any outcomes recommended by the corresponding COS/guidelines. Verbatim guidance document text regarding the suggested outcome measures will be recorded in tabular form for each COS. The matching between the scope of the COS and regulatory guidance will be classified as exact or general (e.g. COS is narrower/broader) in relation to both the population with the condition and the interventions (as per Figure 1), with input from clinical members of the research team and/or the COS developers, if necessary. Data extraction will be carried out by all researchers for the initial three COS/guidance pairs to ensure consistency of approach; subsequent data extraction will be carried out independently by two researchers. Disagreements will be resolved by discussion with SD/PW if necessary. Mapping between core outcomes and outcomes suggested in guidelines will be checked by the lead author (SD).

### Analysis

The mapping of the verbatim extracted text from the EMA/FDA guidance to each of the core outcomes will be coded as specific (i.e. direct correspondence between the wording of the core outcome references in the guidance compared to the wording in COS) or general (i.e. only general alignment between the wording of text in the guidance relative to the wording in the COS), and this mapping will be summarised using a table as demonstrated for the type 2 diabetes SCORE-IT COS ^23^ in Appendix 1. Again, we will contact COS developers if clinical input is required regarding general or specific correspondence between core outcomes and those suggested in the guidance. We will use a tick to demonstrate specific correspondence between the wording of the core outcome references in the guidelines compared to the wording in COS, whereas a tick in brackets will be used to indicate general alignment (with further detail provided in a footnote) between the wording of suggested outcomes in the guidelines relative to the wording in the COS. For each COS, we will record the number (and percentage) of COS outcomes which were covered in the guidance (separately for FDA and EMA) in general or specific terms (separately) and either general/specific terms. The distribution of these percentages will be summarised across guidance documents as a whole, split by FDA and EMA, using descriptive statistics and graphical presentation, overall and split by disease category. We will also present results according to the breakdown of matching between scope of intervention and population between the COS and guidelines, as shown in the matrix in Appendix 2. Note that only results for highlighted cells *a-c*, *e-g*, *i-k* will be presented (i.e. those corresponding to at least a general match in both intervention and population between the COS and guidelines).

In addition, by way of symmetry we will present the results above which instead compare how the core outcomes relate to those suggested in EMA and FDA guidelines, in order to identify the agreement of COS with outcomes suggested in corresponding guidelines; i.e. we will present two additional sets of tables/results, the first with outcomes suggested in the EMA guidelines, and the second with outcomes suggested in the FDA guidelines, as the index list of outcomes. We will explore the impact of COS characteristics (for example, the number of core outcomes or the involvement of LMIC participants in COS development) on their concordance with corresponding EMA/FDA guidelines.

### Dissemination

The findings of this study will be disseminated through publication in an open access peer-reviewed journal and presentation at both national and international conferences. Contact will be made with FDA and EMA colleagues and feedback on our findings requested.

## Discussion

This study will identify any misalignment between outcomes suggested by EMA and FDA regulatory guidance relative to those included in published COS for research, thus demonstrating the degree of representation of core outcomes, which have been agreed by consensus by key stakeholders, within regulatory guidance, and vice versa. A lack of concordance between COS and regulatory guidance may highlight the opportunity for such guidelines to be better informed by COS and vice versa, and we will use the evidence obtained from this study to engage the relevant regulatory bodies in discussions accordingly. We will endeavour to discuss, and ultimately produce guidance about, how researchers should determine key outcomes in the case of lack of concordance between COS and regulatory guidelines.

### Details of working group

ICMJE guidance will be followed with regards to publication policy. 

**Appendix 1.** Tabulated results for SCORE-IT COS (T2D case study ^24^).

SCORE-IT COS	Guidance		SCORE-IT core outcome not explicitly mentioned but covered by the following general terms
	EMA	FDA	
Overall survival			
Death from a diabetes related cause such as heart disease		(✓) ^a^	^a^Cardiovascular disease/safety profile
Heart failure	(✓) ^a^ ^b^	(✓) ^a^ ^c^	^a^Cardiovascular disease/safety profile ^b^Coronary complications ^c^Diabetes-related complications
Gangrene or amputation of the leg, foot or toe	(✓) ^d^	(✓) ^c^	^d^Peripheral vascular diseases ^c^Diabetes-related complications
Hyperglycaemic emergencies ^1^		(✓) ^c^	^c^Diabetes-related complications
Hyperglycaemia		(✓) ^c^	^c^Diabetes-related complications
Hypoglycaemia	✓ ^4^	(✓) ^c^	^c^Diabetes-related complications
Cerebrovascular disease	✓	(✓) ^c^	^c^Diabetes-related complications
Hospital admissions due to diabetes			
Side effects of treatment	✓	✓	
Global quality of life	✓		
Nonfatal myocardial infarction	(✓) ^a^ ^b^	(✓) ^a^ ^c^	^a^Cardiovascular disease/safety profile ^b^Coronary complications ^c^Diabetes-related complications
Visual deterioration or blindness	(✓) ^g^	(✓) ^c^	^g^Retinopathy ^c^Diabetes-related complications
Glycaemic control	✓ ^4^	✓	
Neuropathy ^2^	✓	(✓) ^c^	^c^Diabetes-related complications
Kidney function	✓	(✓) ^c^	^c^Diabetes-related complications
Activities of daily living ^3^	✓		
Body weight	✓ ^4^		

^1^ Including diabetic ketoacidosis and hyperosmolar hyperglycaemic state ^2^ Damage to the nerves caused by high glucose. This can lead to tingling and pain or numbness in the feet or legs. It can also affect bowel control; stomach emptying and sexual function. ^3^ Including those related to personal care; household tasks or community-based tasks. ^4^ Included as core efficacy or safety outcome
