# Supplementary material for: Representation of published core outcome sets for research in regulatory guidance: protocol
Source: HRB Open Res. 2021 Aug 2;4:45. Originally published 2021 May 7. [Version 3] doi: 10.12688/hrbopenres.13139.3 (PMC8311813; doi:10.12688/hrbopenres.13139.3)
Supplement: Peer review history

# Peer review: Reviewer response for version 2

**DOI:** 10.21956/hrbopenres.14512.r29789

**Published:** 2021-07-22

**Authors:** Donna A. Messner

**Affiliations:** 1 Center for Medical Technology Policy, Baltimore, MD, USA

The authors have identified a question that is relevant for researchers, COS developers, and the broader health care community: to what extent do FDA and EMA guidance recommendations align with the results of published core outcome sets? The authors set out a well-conceived protocol for exploring this question in a way that makes relevant comparisons between COS and guidance and reports it coherently. I think this is a project that absolutely needs to be done. 

 In the third paragraph the authors note that uniformity in recommendations from public funders like NIHR would promote greater consistency in outcomes, and “this benefit would have additional impact if the consistency in such recommendations extended to those from commercial sponsors.” Do you mean "for" commercial sponsors?  The statement doesn't make sense to me otherwise.

 The third paragraph notes that FDA and EMA are influential in commissioning research to help inform their decisions. Yes, but in this context it seems much less important to me than their powerful role as arbiters of evidence for commercialization of new products. This is a primary function of their guidance.

 Another thought on impact: FDA and EMA guidance are often not well aligned (to the great frustration of drug developers). So another possible impact of this work might be to facilitate alignment of international regulatory guidance (at least for outcomes) with COS as a common touchstone.

 Also, one technical mistake in the introduction. The fourth paragraph of the intro states that FDA guidance is not legally binding, which is true, but then adds “unlike FDA regulations, which are federal laws.” Regulations are not laws. Laws can only be made by the U.S. Congress. Congress authorizes FDA to write regulations to work out the details of how the laws should be implemented. So regulations are legally binding, but they are not laws. (And there is a missing closed parenthesis at the end of the statement about federal laws.)

Is the study design appropriate for the research question?

Yes

Is the rationale for, and objectives of, the study clearly described?

Yes

Are sufficient details of the methods provided to allow replication by others?

Yes

Are the datasets clearly presented in a useable and accessible format?

Not applicable

Reviewer Expertise:

Expertise in core outcome set development, procedures for medical product regulation and approval in the U.S., health care coverage and reimbursement policy, stakeholder engagement methods, and social/policy research design.

I confirm that I have read this submission and believe that I have an appropriate level of expertise to confirm that it is of an acceptable scientific standard.

# Response

**Published:** 2021-07-23

**Authors:** Susanna Dodd

**Affiliations:** University of Liverpool, UK

In the third paragraph the authors note that uniformity in recommendations from public funders like NIHR would promote greater consistency in outcomes, and “this benefit would have additional impact if the consistency in such recommendations extended to those from commercial sponsors.” Do you mean "for" commercial sponsors?  The statement doesn't make sense to me otherwise.

- **Thank you for pointing out this error – we have changed the word from “from” to “for”. ** 

The third paragraph notes that FDA and EMA are influential in commissioning research to help inform their decisions. Yes, but in this context it seems much less important to me than their powerful role as arbiters of evidence for commercialization of new products. This is a primary function of their guidance.

- **Thank you for this comment - we have added this point in the introduction.**

Another thought on impact: FDA and EMA guidance are often not well aligned (to the great frustration of drug developers). So another possible impact of this work might be to facilitate alignment of international regulatory guidance (at least for outcomes) with COS as a common touchstone.

- **This is an interesting discussion point which we will be covered in the discussion section of the main publication.**

Also, one technical mistake in the introduction. The fourth paragraph of the intro states that FDA guidance is not legally binding, which is true, but then adds “unlike FDA regulations, which are federal laws.” Regulations are not laws. Laws can only be made by the U.S. Congress. Congress authorizes FDA to write regulations to work out the details of how the laws should be implemented. So regulations are legally binding, but they are not laws. (And there is a missing closed parenthesis at the end of the statement about federal laws.)

- **Thank you for pointing out this technical error – we have changed the text “which are federal laws” to “which are the details of how US Congress laws should be implemented”.**

# Peer review: Reviewer response for version 2

**DOI:** 10.21956/hrbopenres.14512.r29639

**Published:** 2021-06-24

**Authors:** Sophie Werkö, Marie Österberg

**Affiliations:** 1 Swedish Council on Health Technology Assessment and Assessment of Social Services (SBU), Stockholm, Sweden; 2 Swedish Agency for Health Technology Assessment and Assessment of Social Services (SBU), Stockholm, Sweden

Thank you, we have now checked the document and have really only one remaining comment for potential improvement.

 We still lack a discussion on the quality of the included COS. We understand if this may be out of scope for this study, but we still believe that it would be interesting to hold such a discussion as we already today are faced with only a little guidance on how to deal with obvious low quality COS. It would be a pity when conducting this otherwise great study, to not include these aspects, because they are already part of the current problems in addressing COS today. Please take this as advice only. We are happy to approve of the current version.

Is the study design appropriate for the research question?

Yes

Is the rationale for, and objectives of, the study clearly described?

Yes

Are sufficient details of the methods provided to allow replication by others?

Yes

Are the datasets clearly presented in a useable and accessible format?

Yes

Reviewer Expertise:

NA

We confirm that we have read this submission and believe that we have an appropriate level of expertise to confirm that it is of an acceptable scientific standard.

# Response

**Published:** 2021-06-24

**Authors:** Susanna Dodd

**Affiliations:** University of Liverpool, UK

Thank you for your comment. We agree with your point and will ensure that we include a discussion in the study publication about the complex issue of COS quality.

# Peer review: Reviewer response for version 1

**DOI:** 10.21956/hrbopenres.14252.r29391

**Published:** 2021-05-24

**Authors:** Sophie Werkö, Marie Österberg

**Affiliations:** 1 Swedish Council on Health Technology Assessment and Assessment of Social Services (SBU), Stockholm, Sweden; 2 Swedish Agency for Health Technology Assessment and Assessment of Social Services (SBU), Stockholm, Sweden

Thank you for letting us review this interesting study. We think the research question is well chosen and will give interesting insights and results of the use and applicability of COS. We only have some minor comments which we hope will be helpful in improving the study further.

1. In the last sentence of the Discussion part in the abstract, the authors state that they will seek feedback from FDA and EMA “if there is a lack of concordance between COS and regulatory guidance overall or for particular disease areas.” We think feedback from the regulatory authorities would be interesting regardless of what the results are.
2. As the COMET database in our understanding includes studies from different countries, we suggest that an example from another country than the UK as well, is included in the introduction to give a broader perspective. As it reads now, there seems to be only the UK or “globally”.
3. Regarding methods, it is stated that the studies should have been published between 2015-2019. Why is 2020 not included (as we are in 2021 now). Please explain why this is: because of COVID-19, nothing was published or any other reason.
4. We currently think there is a lack of discussion on the potential limitations of the study. For example, limitations regarding relevance and context. It would be interesting to see whether a COS -set produced in a high-income country compared to a low-income country differ, and also, if one or the other has influenced EMA or FDA more. We understand that there could be difficulties in drawing conclusions out of this type of information, however it is important to discuss the potential importance of this implication which in the long run may mean that the production of COS will have impact on research outcomes of thus more importance to high-income countries. (Patients or other stakeholders may look upon the prioritization of outcomes differently depending on contextual factors).
5. There is also a lack of discussion on the quality of the included COS. In the section on methods, we think the authors should add a paragraph on how they will assess quality of the included COS. This should be discussed regarding both the selection of participants, how the actual prioritization process is done as well as numbers of the outcomes included in the core outcome set. (Core outcome sets that include large number of outcomes will likely have more of a concordance with regulatory guidance than those with fewer outcomes. It is our opinion that a core set by definition should include less than 10 outcomes, but when it doesn’t this needs to be addressed.
6. It would be interesting if the authors note whether the included published core outcome sets had searched for FDA or EMA documentation regarding important outcomes before they started their study.
7. Finally, we assume the authors will discuss how researchers should relate to core outcome sets that do not include all the requested outcomes requested by regulators. That will be an interesting discussion to read. Looking forward to see this study published!

Is the study design appropriate for the research question?

Yes

Is the rationale for, and objectives of, the study clearly described?

Yes

Are sufficient details of the methods provided to allow replication by others?

Yes

Are the datasets clearly presented in a useable and accessible format?

Yes

Reviewer Expertise:

Health Technology Assessment (HTA)

We confirm that we have read this submission and believe that we have an appropriate level of expertise to confirm that it is of an acceptable scientific standard, however we have significant reservations, as outlined above.

# Response

**Published:** 2021-06-01

**Authors:** Susanna Dodd

**Affiliations:** University of Liverpool, UK

**Thank you for your comments. Our responses are given below:**

- In the last sentence of the Discussion part in the abstract, the authors state that they will seek feedback from FDA and EMA “if there is a lack of concordance between COS and regulatory guidance overall or for particular disease areas.” We think feedback from the regulatory authorities would be interesting regardless of what the results are.

**Thank you for highlighting this point. We agree with your comment and will endeavour to share the results of this study to the regulatory authorities, regardless of the findings.**

- As the COMET database in our understanding includes studies from different countries, we suggest that an example from another country than the UK as well, is included in the introduction to give a broader perspective. As it reads now, there seems to be only the UK or “globally”.

**Thank you for this suggestion. We have added the example of COS endorsement for use in the development of trials and systematic reviews by the Swedish Agency for Health Technology Assessment and Assessment of Social Services (SBU).**

- Regarding methods, it is stated that the studies should have been published between 2015-2019. Why is 2020 not included (as we are in 2021 now). Please explain why this is: because of COVID-19, nothing was published or any other reason.

**This project was initiated in late 2020, and COS identified as part of the annual COMET systematic reviews were included up to and including the systematic review conducted in 2020, which included studies published up to the end of 2019. This explanation has been added to the manuscript.**

- We currently think there is a lack of discussion on the potential limitations of the study. For example, limitations regarding relevance and context. It would be interesting to see whether a COS -set produced in a high-income country compared to a low-income country differ, and also, if one or the other has influenced EMA or FDA more. We understand that there could be difficulties in drawing conclusions out of this type of information, however it is important to discuss the potential importance of this implication which in the long run may mean that the production of COS will have impact on research outcomes of thus more importance to high-income countries. (Patients or other stakeholders may look upon the prioritization of outcomes differently depending on contextual factors).

**Thank you for this suggestion. The vast majority of COS for research are led from a high-income country (HIC); a recent systematic review found only four COS for research that were led from low/middle-income countries (LMIC). As such it is unlikely that it will be possible to make meaningful comparisons between COS from a HIC/LMIC to determine their respective impact on regulatory guidelines. However, the COMET database contains information on whether LMIC participants were involved in COS development, and therefore we could assess whether the involvement of LMIC participants in COS development impacts on the concordance between COS and regulatory guidelines. This has been added to the data extraction and analysis sections of the manuscript.**

- There is also a lack of discussion on the quality of the included COS. In the section on methods, we think the authors should add a paragraph on how they will assess quality of the included COS. This should be discussed regarding both the selection of participants, how the actual prioritization process is done as well as numbers of the outcomes included in the core outcome set. (Core outcome sets that include large number of outcomes will likely have more of a concordance with regulatory guidance than those with fewer outcomes. It is our opinion that a core set by definition should include less than 10 outcomes, but when it doesn’t this needs to be addressed.

**It is relatively difficult to define COS ‘quality’; the COS-STAD standards were introduced in 2017, but we do not think it would be fair or helpful to apply COS-STAD retrospectively to studies that were published before 2017. We decided to include only those COS for research which were published in recent years (between 2015 and 2019) and which involved patients in the consensus process, in order to increase the likelihood that high standard COS are included. We have added to the analysis section that we will explore the impact of the number of core outcomes on the level of agreement between COS and corresponding EMA/FDA guidelines.**

- It would be interesting if the authors note whether the included published core outcome sets had searched for FDA or EMA documentation regarding important outcomes before they started their study.

**Thank you for this comment. This information is currently being extracted from each COS publication, so we have added this explicitly to the methods section.**

- Finally, we assume the authors will discuss how researchers should relate to core outcome sets that do not include all the requested outcomes requested by regulators. That will be an interesting discussion to read. Looking forward to see this study published!

Thank you for this suggestion. This is an interesting point which we had not mentioned in the protocol, so we have now added it to the discussion.
